# Acromioclavicular joint cyst presenting with findings concerning for a soft tissue tumor – a case report

**DOI:** 10.1186/s13000-024-01581-3

**Published:** 2024-12-18

**Authors:** John F. Schutz, Christopher D. Hamad, Michael D. Russell, Mahmuod Abdeljaber, Scott D. Nelson, Fritz Eilber

**Affiliations:** 1 University of Colorado Department of Orthopedics, Aurora, US; 2 Department of Orthopedic Surgery, University of California Los Angeles, Los Angeles , US; 3 Department of Pathology, University of California Los Angeles, Los Angeles, US; 4 Department of General Surgery, University of California Los Angeles, Los Angeles , US

**Keywords:** Cyst, Soft tissue sarcoma, Acromioclavicular joint

## Abstract

**Case:**

We present the case of a 73-year-old female with an acromioclavicular joint cyst associated with atypical, exquisite, progressive pain, and imaging findings concerning for neoplastic etiology. She underwent en bloc resection of the trapezium containing this cystic mass and distal clavicle. Surgical pathology demonstrated findings consistent with a large ganglion cyst without evidence of malignancy.

**Conclusion:**

Our case serves to emphasize the importance of stepwise evaluation and appropriate treatment of such cysts while utilizing appropriate principles of oncologic resection in cases where a neoplastic etiology is considered.

## Introduction

Osteoarthritis of the joints commonly presents with pain, impaired mobility, and occasional formation of associated subchondral cysts, as well as juxta-articular cysts, most commonly seen in the hip, knees, and shoulder joints. Cysts arising from the acromioclavicular joint have been the subject of many case reports, with only 3 reported to have intact rotator cuff musculature [1–3]. Such cysts can raise suspicion for soft tissue tumors, and have previously been called pseudotumor of the shoulder [4]. Hiller et al. first proposed two primary etiologies of AC joint cysts: (1) cysts caused primarily by AC joint degeneration with an intact rotator cuff or (2) cysts associated with AC joint degeneration and massive tears of the rotator cuff [5]. The differential diagnosis for these slow growing masses is broad and can include bone and soft tissue tumors, hematoma, connective tissue and synovial disorders, gout, degenerative joint disease, and infection [5]. 

We present a patient with an unusually painful, multiloculated acromioclavicular joint cyst, with initial imaging findings concerning for soft tissue sarcoma, who was treated with excisional biopsy of the cyst and resection of her distal clavicle.

## Case

A 73-year-old female with history of gout, rheumatoid arthritis, right carpal tunnel, and right hip and left knee arthroplasties presented with a slowly enlarging mass in the area above her left shoulder that was present for several years duration. She had not previously undergone biopsy or any cross-sectional imaging and was referred to surgical oncology for further evaluation and to rule out soft tissue sarcoma as she was concerned about a possible tumor. She denied any recent or remote history of trauma to the area. She endorsed significant pain in the area of the mass. On physical exam, there was a palpable, soft, mobile mass on top of her left shoulder, roughly 5 cm in diameter. The mass was extremely tender to palpation. She had full elbow, wrist, and hand range of motion. Sensation was intact distally. There was no clinical or laboratory evidence of infection.

**Fig. 1:**
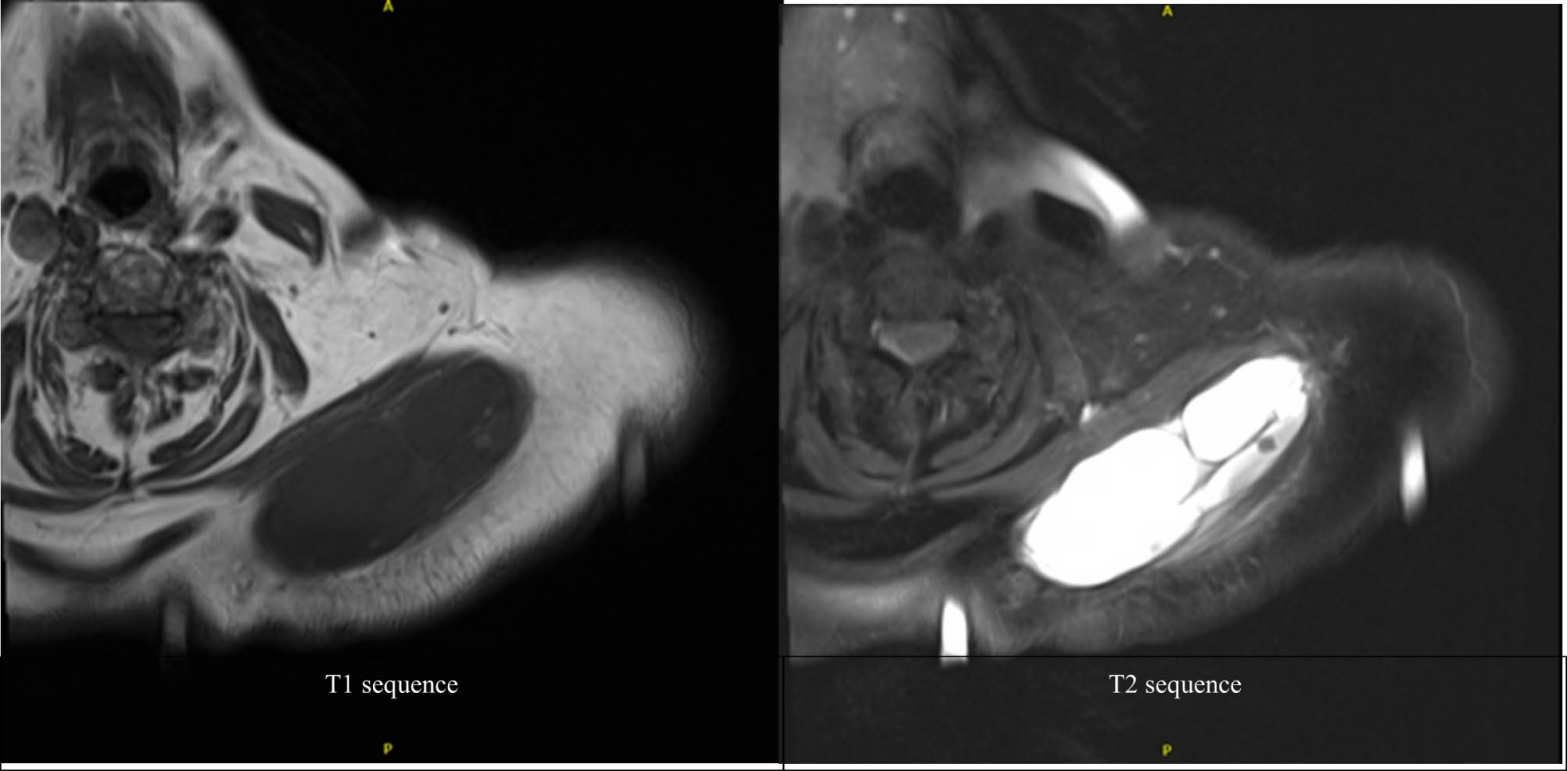
MRI of the cyst demonstrating 10.5 cm x 3.4 cm x 4.4 cm mass originating from the AC joint with peripheral and septal enhancement on T1 and T2 axial sequences

Contrast-enhanced MRI showed a cystic appearing 10.5cm × 3.4cm × 4.4 cm mass originating from the AC joint and extending into the superior fibers of the trapezius muscle. It showed peripheral and septal enhancement. The mass on MRI was hypointense on T1 weighted imaging and hyperintense on T2. (Fig. 1) While the mass was believed to be benign, the complex nature of the cyst coupled with the large size raised concern for possible neoplastic origin of the mass, including soft tissue sarcoma, lymphoma, or schwannoma. MRI also demonstrated diffuse rotator cuff tendinopathy, superior migration of the humeral head, a focus of avascular necrosis of the glenohumeral head with partial collapse, multiple inta-articular bodies, as well as glenohumeral and acromioclavicular osteoarthritis.

The patient underwent en bloc resection of cyst containing portion of trapezius, distal clavicle, and AC joint. The specimen was sent for permanent section. Resection of 0.5 cm of the distal clavicle and AC joint was then performed. Her rotator cuff tear was found to be irreparable and given that it was minimally symptomatic, no cuff repair was pursued. A Blake drain was placed in the resection defect and soft tissue closure was performed. Postoperatively, she was placed in a sling for comfort as needed.

She was seen in clinic on post-op day 8 and day 15. She denied any focal complaints, reported resolution of her pain, and was very satisfied with her outcome. Of note, our patient developed a seroma approximately 1 month post-operatively that required three aspirations. 3 months post-operatively, she underwent IR drain placement, which was maintained for 1 month given continued high output. 4 months post-operatively, this drain was removed and patient’s reaccumulating seroma had resolved. 5 months post-operatively, patient endorsed pain in the operative shoulder and was referred to an orthopedic shoulder and elbow specialist for consideration of arthroplasty. She was indicated for the procedure and is now awaiting a surgical date. There was no clinical or radiographic evidence of recurrence of her cyst at her 5 month follow up.

The patient described in this case was informed that data concerning her case would be submitted for publication and was appropriately consented.

## Pathology

**Fig. 2:**
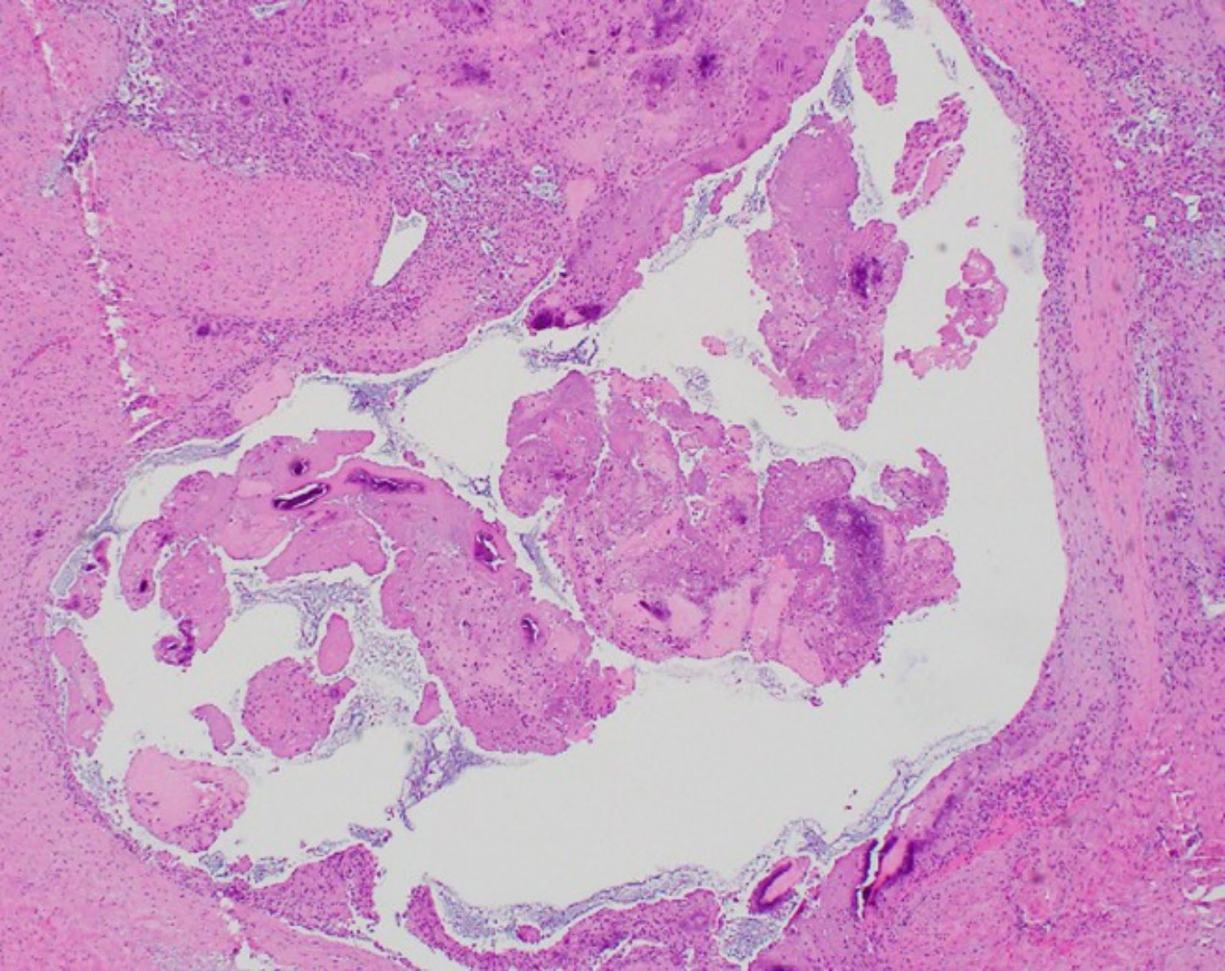
Low-Magnification view of the cyst (Hematoxylin and eosin; 40x magnification)

**Fig. 3:**
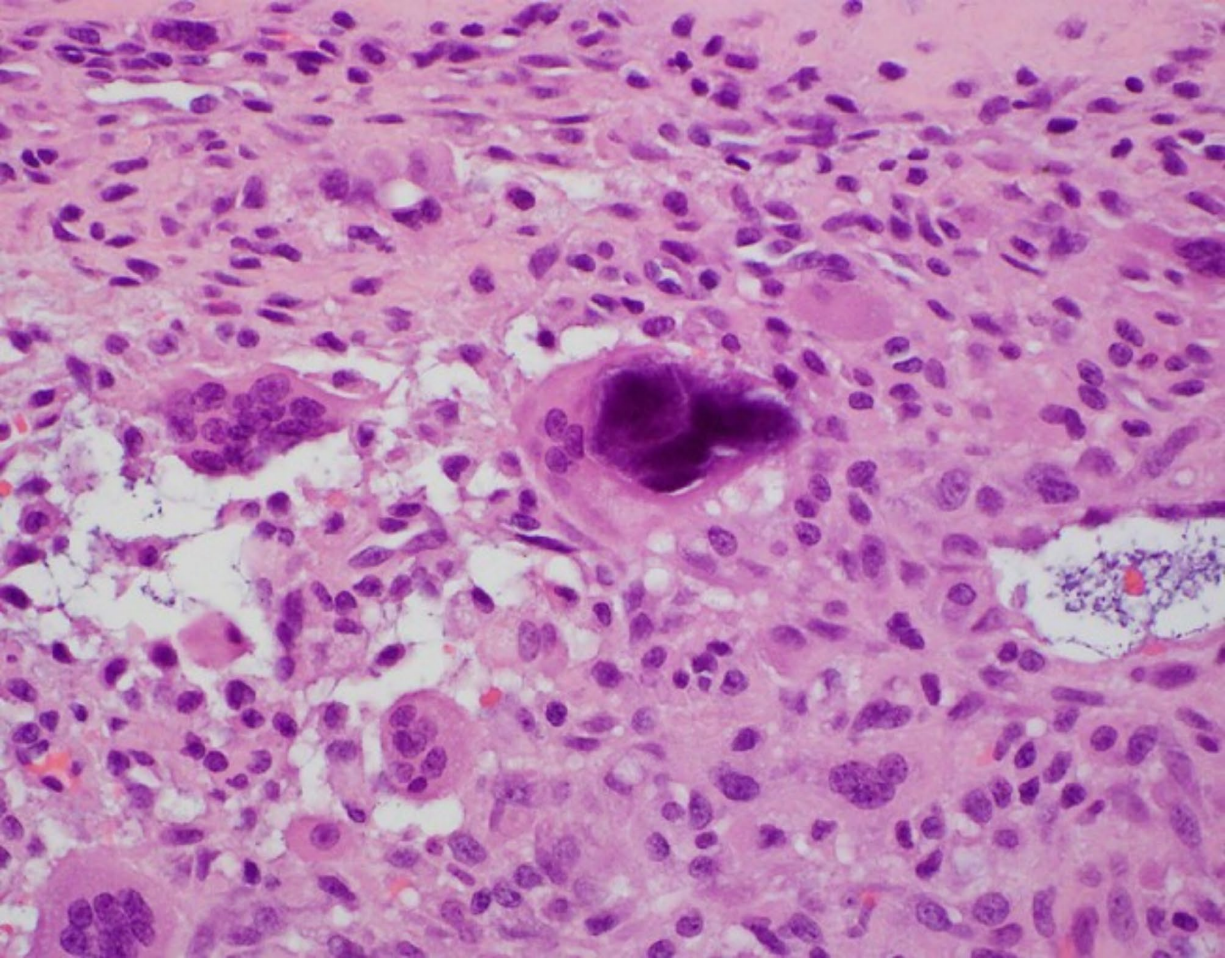
High-Magnification view of calcium pyrophosphate dihydrate crystals and associated giant cell reaction (Hematoxylin and eosin; 400x magnification)

Gross examination of the en bloc resection of the cyst revealed a cystic structure exhibiting a partially solid and partially multiloculated structure, measuring 5.8 cm at its greatest dimension. Sectioning revealed yellow-tan to tan-red mucoid to gelatinous material. The solid components were white-tan and semi-firm, attached to skeletal muscle. Low power microscopic examination revealed an unlined cyst with abundant fibrin, myxoid degeneration, and notable calcification, accompanied by a giant cell reaction (Fig. 2). Examination of the calcifications at higher magnification showed collections of crystalline, polygonal material, consistent with calcium pyrophosphate dihydrate, alongside an associated giant cell reaction (Fig. 3). Examination of the distal clavicle demonstrated lamellar bone, fibrocartilage, and marrow with trilineage maturation. There was no pathologic evidence of malignancy in either the soft tissue or bony specimen.

## Discussion

Acromioclavicular joint cysts are rare complications of AC joint degenerative disease and rotator cuff tears. Two distinct etiologies of AC joint cysts have been described in the literature [5]. Cysts limited to the AC joint can arise as a result of isolated AC joint degeneration with an intact rotator cuff. In the setting of massive rotator cuff tears, cuff tear arthropathy can cause upward displacement of the humeral head with associated erosion into the AC joint space. This then allows glenohumeral joint synovial fluid to accumulate in the AC joint, distending the superior capsule of the AC joint, and causing the appearance of a large soft tissue mass [1, 5]. 

These cysts most commonly appear in elderly patients without any recent or remote history of trauma to the area. The differential diagnosis for such soft tissue masses in the area of the AC joint is broad and includes infectious etiologies, hematoma, and bone or soft tissue tumors [6]. Cysts are almost always reported to be painless and rotator cuff tears are often well compensated [7, 8]. The patient in our case presented with exquisite pain and tenderness of the mass, which is atypical for these lesions and raised suspicion for a possible neoplastic etiology.

Initial evaluation of masses around the AC joint should include a careful physical examination, including an exam of the rotator cuff to determine the need for rotator cuff repair or reverse total shoulder arthroplasty. Imaging initially includes plain radiographs and MRI to characterize the lesion. Arthrogram was the historical imaging modality of choice. Craig first described the pathognomonic “geyser sign” seen on arthrogram, wherein dye injected into the glenohumeral joint flows into the subdeltoid bursa and into the cyst, creating the appearance of a geyser [9]. Radiographs most often show cuff tear arthropathy and AC joint degeneration, as well as soft tissue swelling in the area of the mass. MRI, however, is most often used for further evaluation and AC joint cysts appear as homogenous, hypointense on T1 and hyperintense on T2, often with evidence of fluid communication between the glenohumeral and AC joints [3, 10]. Some authors are now suggesting point-of-care ultrasound (PoCUS) as a simple, cost-effective, low-risk method for characterizing nonspecific soft tissue swelling and narrowing the differential diagnosis [11]. In our reported case, MRI of the cyst demonstrated septations of the lesion which again raised concern for a possible neoplastic cause for the patient’s symptoms.

Management of AC joint cysts remains controversial and numerous operative and non-operative methods have been described in the literature. Treatment options for Type 1 and 2 AC joint cysts include aspiration, distal clavicle resection and cyst excision, arthroscopic irrigation and debridement, total shoulder arthroplasty, and reverse total shoulder arthroplasty [2, 6, 12, 13]. Aspiration most commonly results in recurrence and presents a risk for infection or fistula formation [1, 4, 6]. 

In our described case, the patient was concerned about the appearance of the mass and the pain it caused her. Her cuff-tear arthropathy was well compensated and minimally symptomatic. We elected to pursue distal clavicle excision due to the significant size of the cyst, irreparable and asymptomatic nature of her rotator cuff tear, and concern for possible neoplastic etiology. This method also allowed for wide excision of the lesion in the event that surgical pathology was consistent with malignancy. Similar approaches to address AC joint cysts without rotator cuff repair or shoulder arthroplasty have been reported to be successful, including arthroscopic debridement and cyst excision or distal clavicle excision with allograft or collagen patching when joint capsule tissue is irreparable [6, 7, 13]. Recurrence of the cyst after resection of the AC joint or after rotator cuff repair is rare [7, 10, 13, 14]. 

Our case describes a patient who presented with a cyst of the acromioclavicular joint with initial concern for neoplastic etiology who was treated with en bloc resection of the cyst and distal clavicle. The benign nature of the cyst was confirmed by surgical pathology and histologic examination. Our case serves to emphasize the importance of performing a stepwise workup in evaluation of an atypical presentation of an acromioclavicular cyst and employing oncologic resection principles in the resection of such a cyst when etiology of the lesion is uncertain.

## Data Availability

No datasets were generated or analysed during the current study.
